# Extensive Translatome Remodeling during ER Stress Response in Mammalian Cells

**DOI:** 10.1371/journal.pone.0035915

**Published:** 2012-05-04

**Authors:** Iván Ventoso, Alex Kochetov, David Montaner, Joaquín Dopazo, Javier Santoyo

**Affiliations:** 1 Departamento de Biología Molecular, Universidad Autónoma de Madrid and Centro de Biología Molecular Severo Ochoa (UAM-CSIC), Cantoblanco, Madrid, Spain; 2 Department of Bioinformatics and Genomics, Centro de Investigación Príncipe Felipe (CIPF), Valencia, Spain; 3 CIBER de Enfermedades Raras (CIBERER), ISCIII, Valencia, Spain; 4 Functional Genomics Node (INB), CIPF, Valencia, Spain; 5 Institute of Cytology and Genetics, Novosibirsk, Russia; North Carolina State University, United States of America

## Abstract

In this work we have described the translatome of two mammalian cell lines, NIH3T3 and Jurkat, by scoring the relative polysome association of ∼10,000 mRNA under normal and ER stress conditions. We have found that translation efficiencies of mRNA correlated poorly with transcript abundance, although a general tendency was observed so that the highest translation efficiencies were found in abundant mRNA. Despite the differences found between mouse (NIH3T3) and human (Jurkat) cells, both cell types share a common translatome composed by ∼800–900 mRNA that encode proteins involved in basic cellular functions. Upon stress, an extensive remodeling in translatomes was observed so that translation of ∼50% of mRNA was inhibited in both cell types, this effect being more dramatic for those mRNA that accounted for most of the cell translation. Interestingly, we found two subsets comprising 1000–1500 mRNA whose translation resisted or was induced by stress. Translation arrest resistant class includes many mRNA encoding aminoacyl tRNA synthetases, ATPases and enzymes involved in DNA replication and stress response such as BiP. This class of mRNA is characterized by high translation rates in both control and stress conditions. Translation inducible class includes mRNA whose translation was relieved after stress, showing a high enrichment in early response transcription factors of bZIP and zinc finger C2H2 classes. Unlike yeast, a general coordination between changes in translation and transcription upon stress (potentiation) was not observed in mammalian cells. Among the different features of mRNA analyzed, we found a relevant association of translation efficiency with the presence of upstream ATG in the 5′UTR and with the length of coding sequence of mRNA, and a looser association with other parameters such as the length and the G+C content of 5′UTR. A model for translatome remodeling during the acute phase of stress response in mammalian cells is proposed.

## Introduction

Gene expression is regulated at multiple levels to adjust the concentration of macromolecular components to the physiological demands of the cell and organism. The amount of a given protein in the cell depends not only on the transcriptional activity of its gene, but also on the balance between post-transcriptional and post-translational processes that affect the synthesis and stability of the protein [1], [2], [3], [4]. For years, the relevance of translation in the control of gene expression outputs has been underestimated and restricted to a few examples of mRNA that undergo extreme cases of translation control [5], [6], [7], [8]. However, the discovery that the activities of key translation initiation factors such as eIF2 and eIF4F are tightly regulated by environmental stress and by mitogenic or developmental signals, definitely changed our view of translation control in mammalian cells [9], [10], [11], [12], [13], [14], [15], [16]. More recently, the discovery of widespread changes in protein synthesis induced by microRNAs further supported the key role of post-transcriptional steps of mRNA in gene expression control [17], [18], [19]. Initiation is the limiting step of protein synthesis and the most important control point in eukaryotic translation. Collectively, the activity of eIF4F complex promotes the recruitment of mRNA to ribosomes via cap recognition and scanning to reach the initiation codon [14], [20], [21]. At this last step, the activity of eIF2 is essential for delivering the Met-tRNA_i_ to the 40 S ribosome that promotes codon-anticodon base pairing on AUG triplet during initiation. The activity of eIF2 is blocked by phosphorylation at the S51 of the alpha subunit (eIF2) that prevents the normal recycling of this factor necessary for ongoing translation of most mRNA in the cell [7], [22]. Four stress-activated kinases phosphorylate eIF2 in response to a wide variety of stresses resulting in an almost instantaneous halt of general translation necessary for a effective response to stress [7], [23], [24]. Apart from this general regulation, specific features in mRNA such as the presence of cis-acting sequences and structures in the 5′- and 3′-UTRs, together with the context of initiation codon (AUG) can influence the rate of translation initiation of particular mRNA [25], [26], [27], [28], [29]. Extensive secondary structure in the 5′UTR can prevent ribosome recruitment or scanning in some mRNA, but not in others that initiate by binding of the ribosomes to internal structures within 5′UTR (e.g viral IRES) [14], [21], [30], [31], [32]. The recognition of initiator AUG by the 40 S ribosome also requires an optimal sequence context (A/Gnn**AUG**G) that has been found in most of murine and human mRNA [25], [26]. However, under suboptimal context a fraction of 40 S can skip initiation codon and continues scanning in 3′ direction to initiate at downstream AUG (leaky scanning). The presence of AUG triplets upstream the initiation codon can also influence the rate of translation initiation when eIF2 is available in the cell. The paradigmatic example of this control operates on ATF4 mRNA that encodes a master regulator of stress response in both vertebrate and yeast cells (called GCN4) [5], [13], [33], [34]. Under optimal conditions, upstream short ORFs in 5′UTR are occupied by 40 S ribosomes that after translating short peptides do not resume scanning to reach the downstream, authentic initiation codon of ATF4. Stress-induced phosphorylation of eIF2 relieves translation repression of ATF4 mRNA by promoting reinitiation at the authentic mRNA [5], [33]. A similar mechanism of translation activation during stress has been described recently for transcription factors ATF5 (that also belongs to the ATF/CREB family), for CHOP that acts as an effector of ATF4 and for GADD34 that promotes eIF2α dephosphorylation and translational recovery after stress [35], [36], [37], [38]. However, more mammalian mRNA is suspected to be translated by an ATF4-like mechanism during stress response [39], [40].

Recently, a new concept of coordinated post-transcriptional regulation of mRNA subsets (RNA regulons) by RNA-binding proteins (RBPs) has emerged to integrate translation into a superior level of regulation coupled to splicing, transport and mRNA stability [41].

The implantation of high throughput analysis such as microarrays and, more recently, RNAseq has allowed a deeper understanding of how yeast cells adapt their translation and transcription to environmental changes such as nutrient deprivation or chemical stresses [42], [43], [44]. A such as wide analysis, however, has not been performed yet for mammalian cells so that our current understanding of how mammalian translatome adapts to stressful situations is still partial [10], [19], [45], [46], [47], [48], [49]. The fact that response of mammalian cells to the environmental changes is under the dictatorship of tissues and organs predicts a number of differences with yeast. In this work we used polysome profiling coupled to microarray detection to catalogue the translational efficiencies of murine and human mRNA, in control and in cells subjected to endoplasmic reticulum (ER) stress.

## Results

### Scoring the Translation Efficiencies of Mammalian mRNAs

**Figure 1:**
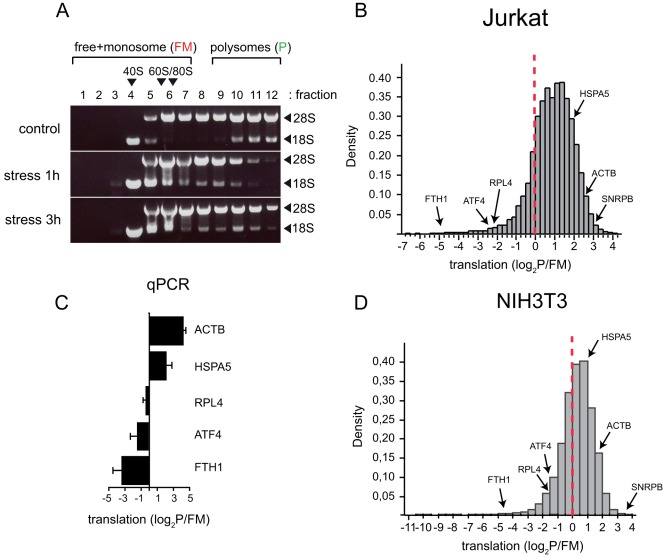
Translatomes of NIH3T3 and Jurkat cells based on polysome profiling. A) The quality of polysome preparation was verified by electrophoretic analysis of RNA content in each fraction. The effect of thapsigargin treatment (stress) for the indicated hours on polysome distribution in Jurkat cells is also shown. A comparable result was obtained in NIH3T3 cells (Figure S1). Ribosomal 18 S and 28 S bands are shown. B) and C) Density distribution of translation efficiencies of 10,670 coding mRNAs from Jurkat and 8,459 mRNAs from NIH3T3 under optimal growth conditions. Those mRNA equally distributed among FM and P fractions showed a log_2_P/FM = 0 (marked by a dotted red line). The median values for Jurkat and NIH3T3 cells were 1.07 and 0.45, respectively. The translation efficiencies of some representative mRNA are shown (ACTB, HSPA5, SNRPB, RPL4, ATF4 and FTH1). D) qPCR validation of translation efficiencies for some of the above mentioned mRNA. Data are the mean ±SD from two independent qPCR reactions in Jurkat and NIH3T3 cells.

To catalogue the translation efficiencies of mammalian mRNAs in growing cells, we quantified the fraction of mRNAs engaged or not in translation by means of polysome profiling followed by dual-color microarray analysis. We chose two cell lines for this purpose: murine fibroblasts (NIH3T3) and human leukemia T cells (Jurkat). These two cell lines show remarkable differences in lineage, degree of transformation and substrate adherence, although the rates of proliferation observed during the course of the experiments were similar for both cell types. RNA analysis of fractionated sucrose gradients confirmed the good separation of light fractions (RNPs and monosomes) from heavy ones containing large polysomes (Figure 1A and Figure S1). In parallel, we also treated cell cultures with 10 µM of thapsigargin, a well known stressor that induces an unfolded protein response (UPR) in mammalian cells by disrupting ER homeostasis [13], [50]. This triggers the activation of PERK that phosphorylated eIF2α leading to a general inhibition of cellular translation. As expected, stress induced the disaggregation of heavy polysomes and the accumulation of material in monosome fractions, a typical sign of initiation blockade [20]. An equivalent result was obtained in NIH3T3 cells (Figure S1). RNA from pooled fractions of free+monosome (FM) and polysome (P) cuts were labeled using Cy5 (FM) and Cy3 (P) dyes for competitive hybridization of the chips. The translation efficiency of a given mRNA was defined as the log_2_ ratio of Cy3/Cy5 signals that represents the fraction of mRNA bound to polysomes (translating). Thus, each mRNA located in the P fraction contain two or more engaged ribosomes whereas the mRNA in FM fraction contain one ribosome at most. Using Agilent 44K chips, we were able to detect the expression of 12,500 RNA sequences in Jurkat and 10,500 in NIH3T3 cells with technical reliability. From them, 10,670 sequences in Jurkat and 8,459 in NIH3T3 cells were coding mRNAs. Density distribution showed that most of the mRNA (67% of NIH3T3 and 84% of Jurkat) were enriched in the polysome fractions with log_2_P/FM≥0. However, the median of translation efficiencies was significantly higher in Jurkat cells (1.07 versus 0.45) reflecting an accelerated rate of translation typical of tumor cells [51], [52], [53]. To validate our technical approach, we calibrated the density distributions with mRNAs whose translational behavior has been experimentally documented. First, we chose ß-actin (ACTB) and GRP78 (HSPA5) as representative members of fast and mild translating mRNA, respectively, based on previous data [54], [55]. We also included in this class the mRNA encoding the splicing component SNRNPB that showed one of the highest log_2_P/FM scores in our analysis (3.6 for NIH3T3 and 3 for Jurkat). In the opposite side, the heavy chain of ferritin receptor (FTH1) and ATF4 were selected as representative members of those mRNA subjected to an intense translational repression under growth conditions [13], [56]. In this group we also included the ribosomal protein L4 (RLP4) mRNA that showed a low log_2_P/FM value (∼ −2). As shown in Figure 1B and 1D, FTH1 and ATF4 fell into the bottom 2.5% percentile in Jurkat and into the 5% in NIH3T3 cells with log_2_ ratios below −2 and −1.5, respectively. In the opposite side, ß-actin and GRP78 fell into the top 10% percentile with log_2_P/FM above 1.5 in both cells types. These results were validated by qRT-PCR on the same fractions used for microarray hybridizations (Figure 1C). The consistency found supports the validity of our approach and the quality of polysome profiling used for the analysis.

The wide range of translation efficiencies found among mRNA in both cell types could be reflecting wide differences in mRNA abundance. To test this, we plotted the log_2_ P/FM values with the corresponding A values for each mRNA that was indicative of abundance (see materials and methods). Poor correlation coefficients were obtained (R^2^<0.1 for both mouse and human cells) although a general tendency was observed so that mRNA with the higher A values (from 10 up to 17) tended to show positive log_2_P/FM values (Figure S1). However, this trend was abruptly lost in a subset of very abundant mRNA that showed very low translation coefficients (Figure S1). Thus, the high abundance of a given mRNA does not necessary leads to an efficient translation.

**Figure 2:**
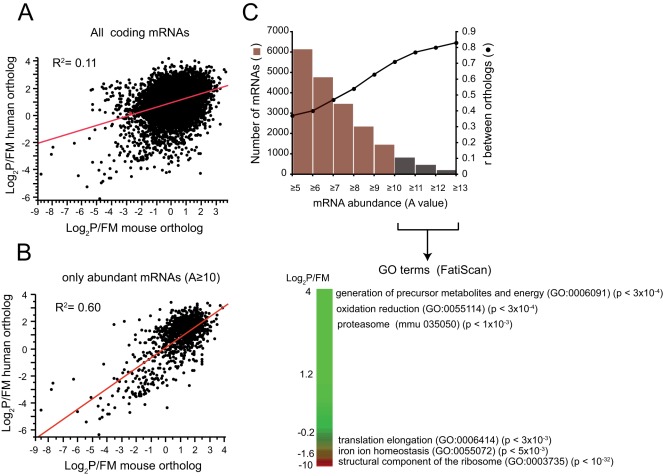
Defining the basic translatome shared by NIH3T3 and Jurkat cells. A) and B) Correlation analysis of log_2_P/FM values among all mouse-man ortholog mRNA pairs detected in the chips (8,019) or among those that showed the highest abundance (A value ≥10; 802 mRNAs). Only coding mRNA were used. Note the significant increase in R^2^. C) A more detailed analysis of the correlation between mRNA abundance and translational consistency among orthologs. Note that pearson’s correlation coefficients (r) increased proportional to mRNA abundance. The subset of mRNA orthologs showing A values ≥10 was subjected to functional analysis using the FatiScan programme. Translation efficiencies were ranked in green to red according to NIH3T3 data and the most significant GO and KEEG terms enriched along the ranking were annotated on the right together with the adjusted p values. A similar result was obtained when the list was ranked according to Jurkat data. Note the specific enrichment in GO terms for orthologs with top and bottom log_2_P/FM values.

Despite the significant differences between Jurkat and NIH3T3 cells, we looked for the existence of a common translatome using the ortholog mRNA pairs that showed comparable translational efficiencies in both cell types. Regression analysis performed with the full list of human-mouse coding mRNA orthologs revealed a poor correlation in log_2_P/FM values (Pearson’s r = 0.37, R^2^ = 0.11) that significantly increased when the analysis was restricted to abundant mRNA. Thus, for mRNA with A values higher than 10 (that fell into the top 10% percentile of abundance), we found a significant correlation (Pearson’s r = 0.73, R^2^ = 0.60) of translation efficiencies among orthologs (Figures 2A and 2B). These results show that Jurkat and NIH3T3 cells share a basic translatome that is composed by the most abundant mRNA (about 800 mRNAs). To test whether this group of mRNA showed a coherent functional meaning, we analyzed the enrichment in Gene Ontology (GO) terms using the FatiScan programme [57] that allowed us to scan the ranked list of mRNA according to log_2_ P/FM values by means of partitions. Thus, orthologs with the highest translation rates were enriched in proteasome components, enzymes involved in the generation of precursor metabolites and energy and oxidation-reduction metabolism. The orthologs with the lowest translation rates were highly enriched in some components of ribosome and translation elongation factors (Figure 2C). A similar enrichment in these GO terms arose when FatiGO analysis [57] was carried out using the selected group of high and low translation mRNA for human and mouse separately. Interestingly, this analysis also revealed the existence of cell-specific mRNA subsets with a specific enrichment in GO terms. For instance, the high translation group of mRNA in NIH3T3 was also enriched in GO terms as RNA splicing (GO:0008380) and response to oxidative stress (GO:0006979), whereas in Jurkat cells the high translation group were also highly enriched in GO terms as DNA replication (GO:0006260), protein folding (GO: 0006457), response to DNA damage stimulus (GO:0006974), mRNA splicing (GO:0008380) and Golgi vesicle transport (GO:0048193) (Figure S2).

We next searched for any association between translation efficiencies and basic features in mRNAs. The parameters analyzed included the length and G+C composition of CDS, 5′ UTR and 3′UTR regions, the presence of upstream ORFs and the context around the initiation and termination codons, as well as the base pairing probability (BBP) distribution around the initiation and termination codons as a rough estimate to the existence of stable RNA secondary structures. All these parameters have been described before to affect translation of specific mRNAs [26], [58], [59], [60], [61]. When full lists of mRNAs were used, no correlation of translation efficiencies with none of parameters analyzed was found (all R^2^<0.1), neither for mouse nor for human cells. So, we next focused on those mRNAs that showed the highest and the lowest translation efficiencies. We analyzed mouse and human mRNAs separately and the results are summarized in Table 1. First, mRNA with high (and to a lesser extent, low) translation efficiencies showed a shorter CDS as compared with the rest of mRNA. For NIH3T3 cells, mRNA with the highest translation rates encode proteins that are about half the size of the rest. The group of mRNA with low translation rates also showed a slight shorter CDS, especially in Jurkat cells (Table 1). Second, in NIH3T3 cells the high translation group showed a significantly shorter 5′UTRs and 3′UTRs. This trend, however, was not observed in Jurkat cells (see discussion). Third, in the high and low translation groups of mRNA the presence of upstream AUG in the 5′UTR was clearly under- and over-represented, respectively, being this correlation much more apparent in NIH3T3 than in Jurkat cells (see discussion). Moreover, the group of mRNAs with low translation efficiencies showed a lower G+C content in the 5′UTR. No significant differences were found in the context of initiation codon (RnnATG) or termination codon usage. We also compared the positional base pair probability (BBP) distribution at the beginning and end of CDS among mRNA with high and low translation rates in NIH3T3 cells. Two stretches of BBP, one located at 18–26 nts downstream the initiation codon and the other located at 13–15 nts downstream the stop codon appeared enriched in the high translation group of mRNA (Figure S2). This could suggest the existence of RNA hairpins (or another element of secondary structure) that could improve the recognition of initiation and stop codons in mRNA with high translation rates as described before for some viral mRNA [28], [62].

**Table 1:** Features of mRNA grouped in translation classes.

NIH3T3					
		Translation class			
	Total	High	Low	Resistant	Inducible
number of mRNAs*	8159	317	383	276	599
CDS length (nts)	1257	**801**	**939**	1506	**2241**
CDS G+C (%)	53,1	53,96	**51,54**	53,33	**48,59**
RnnAUG frequency	0,87	0,91	0,86	0,89	0,84
5′ UTR length (nts)	144	**105**	133	**191**	**206**
5′UTR G+C (%)	65,31	64,14	**61,66**	**61,49**	**61,48**
3′UTR length (nts)	804	**298**	**586**	973	**1529**
3′ UTR G+C (%)	43,95	45,2	**39,46**	45,26	**38,85**
uAUG≥1 (%)	43,28	**19,56**	**51,17**	**38,08**	**58,93**
uAUG≥2 (%)	22,36	**7,26**	**29,5**	19,6	**36,4**
uAUG ≥3 (%)	13,3	**4,11**	**18,75**	12,35	**24,05**
uAUG≥4 (%)	7,88	**1,9**	**11,96**	6,92	**15,32**
uAUG≥5 (%)	4,77	**1,9**	**8,87**	4,75	**10,14**
**Jurkat**					
		**Translation class**			
	**Total**	**High**	**Low**	**Resistant**	**Inducible**
number of mRNAs*	10303	524	426	1330	166
CDS length (nts)	1317	**1055**	**891**	**1968**	**2225**
CDS G+C (%)	53,8	**46,64**	**56,46**	**47,96**	**48,51**
RnnAUG frequency	0,86	0,87	0,88	0,86	0,84
5′ UTR length (nts)	174	188	**127**	**227**	178
5′UTR G+C (%)	66,51	68,12	**63,29**	**65,19**	**60,53**
3′UTR length (nts)	847	**1222**	**352**	**1703**	697
3′ UTR G+C (%)	41,98	**35,34**	42,55	**38,03**	**40,71**
uAUG≥1 (%)	48,96	**45,6**	**43,9**	**58,12**	**41,57**
uAUG≥2 (%)	27,07	**23,65**	**23,24**	**34,74**	25,3
uAUG≥3 (%)	16,74	15,25	15,05	**22,26**	18,07
uAUG≥4 (%)	10,54	10,68	12,2	**15,94**	**13,86**
uAUG≥5 (%)	7,06	6,48	8,44	**10,38**	7,23

* Only mRNAs with A values ≥5.5+5′ UTR≥10+3′ UTR≥10 were selected for the analysis.

High = log2P/FM≥2. Low = log2P/FM≤−1,5. For rows 2, 5 an 7, values are medians. For the rest, values are expressed in % or frequency. Highly significant differences in the t-test are marked in bold (p<0.005).

### Translational Changes Upon Stress

**Figure 3:**
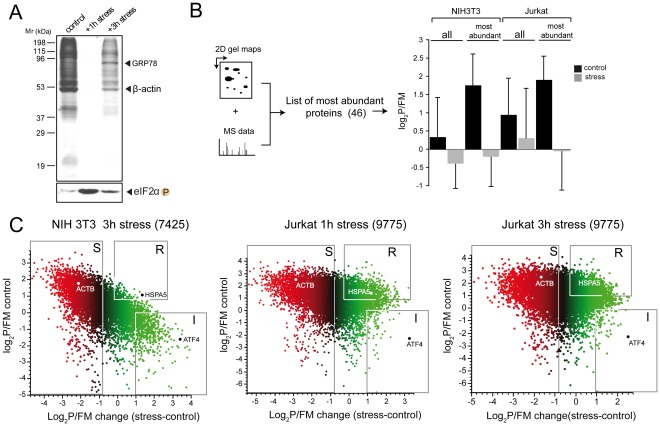
Stress-induced remodeling of translatomes. Identification of translation classes of mRNA. A) Analysis of the translation change after stress using the classical metabolic labeling of newly-made proteins with [^35^S]-Met. NIH3T3 cells were treated with thapsigargin for the indicated times, labeled for 30′ and proteins were analyzed by SDS-PAGE and autoradiography. Note that [^35^S]-Met incorporation was inhibited by 90% at 1h and by 70% at 3 h after stress treatment. The phosphorylation state of eIF2α was analyzed by western-blot in parallel (lower panel). B) The synthesis of proteins that account for most of cellular translation was preferentially inhibited after stress. Based on 2D PAGE analysis and mass spectroscopy (MS) data extracted from the literature, we built a list with the 46 most abundant proteins found in NIH3T3 and Jurkat cells (see Table S1). The mean ±SD of log_2_P/FM values for this mRNA subset under control and stress conditions are shown and compared with values obtained for all mRNA in both cell types. C) Plots showing the change in translation efficiencies (log_2_P/FM stress-log_2_P/FM control) after thapsigargin treatment (3 h for NIH3T3, 1h and 3 h for Jurkat). In parentheses are the number of mRNA used in the analysis. Quadrants were set to identify the translation classes according to values in log_2_P/FM change upon stress. The sensitive (S) class comprises mRNA whose translation decreased≥0.8 log_2_ (40–50% of mRNAs in both NIH3T3 and Jurkat). A representative member of this group is the ACTB mRNA. Resistant (R) class includes those mRNA that continue to translate at moderate to high rates during stress. These mRNA show a log_2_P/FM≥0.8 in both control and stressed cells, and comprises about 3–4% of total mRNA in NIH3T3 and up to 13% in Jurkat cells. A representative member of this group is the HSPA5 (the BiP chaperone) mRNA. Translation inducible class (I) comprises mRNA with low translation efficiencies under control conditions (log_2_ P/FM≤0) that increased upon stress (log_2_ change≥1). This group comprises about 8% of mRNA in NIH3T3 cells and 1.5% in Jurkat cells. A representative member of this group is the transcription factor ATF4.

After treatment of cells with thapsigargin, a rapid halt of protein synthesis associated to eIF2 phosphorylation was observed [50]. When this phenomenon was quantified by [^35^S]-Met incorporation into newly-made proteins, the translational blockade observed was close to 90% during the first hours of treatment (Figure 3A). After 3–4 hours, protein synthesis began to recover due to progressive eIF2α dephosphorylation as described previously [23], [63]. When we analyzed by polysome profiling the global changes of translation efficiencies after stress, we found that about 50% of mRNAs in both NIH3T3 and Jurkat cells showed a significant (≥0.8) decrease in log_2_ P/FM. These apparently discordant results could be explained by the fact that those mRNA that mostly contribute to the translation activity of the cell could be those that experienced the strongest translational repression during stress. To confirm this possibility, we constructed a dataset with the most abundant proteins found in NIH3T3 and Jurkat cells based on previous proteomic analysis using 2D PAGE and mass spectroscopy (MS) data [64], [65], [66], [67]. This list comprises 46 abundant proteins including cytoskeletal components (ß-actin and tubulin ß2), proteasome subunits, and metabolic enzymes such as lactate and malate dehydrogenases among others (Table S1). As predicted, translation of most of these mRNA was strongly blocked after stress (Figure 3B). Functional analysis of mRNA that experienced the strongest translational repression showed GO terms such as electron transport chain (GO: 0022900, p<1.4×10^−5^) and proteasome complex (GO: 0000502, p<1.21×10^−11^) in both cell types.

**Figure 4:**
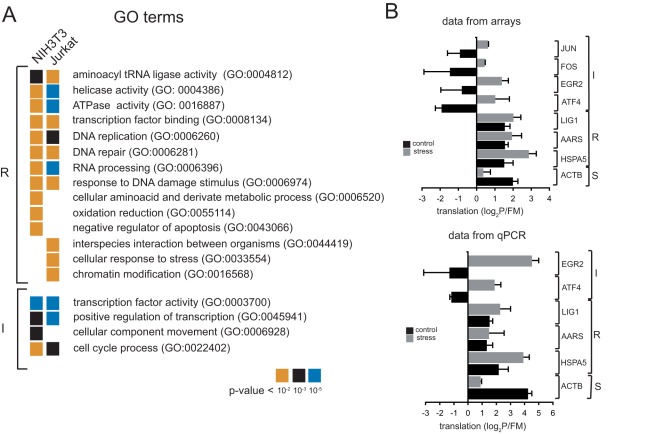
Validation and functional analysis (GO) of translational classes. A) GO terms found in translation classes. Only the most enriched terms with adjusted p-values<10^−2^ are shown (FatiGO analysis). Note the existence of some cell-specific terms in NIH3T3 and Jurkat cells. B) Translational changes upon stress of some representative mRNA of S, R and I classes detected by microarrays. For R class, we focused on mRNA encoding HSPA5 (BiP), the alanyl-tRNA synthetase (AARS) and the ligase I (LIG1). For I class, in addition to ATF4, we also analyzed the mRNA encoding the transcription factors early growth response protein 2 (EGR2) and the proto-oncogenes c-Fos (FOS) and c-Jun (JUN). The ACTB mRNA is a representative member of S class. Lower panel shows the validation of log_2_P/FM data by qPCR for some mRNA.

An examination of the plots of Figure 3C revealed that, in addition to mRNAs whose translation was significantly reduced (stress sensitive, S), a relatively abundant group of mRNA showed no change or positive changes in translation coefficients after stress. Accordingly, we named these groups as translation arrest resistant (R) and translation inducible (I), respectively. The R group of mRNAs showed positive translation values (log_2_ P/FM≥0.8) in both control and thapsigargin-treated cells and comprises about 300 mRNAs (3–4% of total) in NIH3T3 and about 1,360 mRNAs (13%) in Jurkat cells. A representative member of this group is the HSPA5 gene encoding the BiP chaperone, whose mRNA showed relatively high rates of translation in both control and stressed NIH3T3 and Jurkat cells (Figure 3C). Gene ontology (GO) analysis revealed that translation arrest resistant class of mRNAs was enriched in functional terms such as aminoacyl tRNA ligase (GO:0004812), transcription factor binding (GO:0008134), DNA replication (GO:0006260) and repair (GO:0006281), negative regulator of apoptosis (GO:0043066) and RNA processing (GO: 0006396) among others (Figure 4A). Interestingly, when we focused on R mRNAs in Jurkat cells, GO terms related to DNA replication (GO:0006260) or cellular response to stress (GO: 0033554) were also specifically enriched (Figure 4A). The translational resistance to stress for some members of this class of mRNA such as AARS (alanyl tRNA synthetase), Lig1 (DNA ligase 1) and HSPA5 (BiP chaperone) were also confirmed by qPCR (Figure 4B).

Translation inducible group (I) of mRNA is represented by ATF4 and includes about 672 mRNAs in NIH3T3 (8% of total) and 189 mRNA in Jurkat cells (1.5% of total). Similar to ATF4 mRNA, translation of these mRNA was transiently relieved after stress, being further repressed at later times. This behavior contrasted with that observed for stress resistant mRNA that were efficiently translated in both control and stress situations. For I class mRNA, gene ontology analysis revealed a high enrichment in transcription factor activity (GO:0003700) and positive regulation of transcription (GO:0045941) (Figure 4A). Indeed, apart of ATF4, many other transcription factors such as EGR1 and EGR2, NFAT5, ATF5, JUN, FOS, CREB1, OCT1 and SP1 were translationally induced after stress in both cell types (Figure S3). This I class of mRNA is also highly enriched in other many zinc finger and bromodomain-containing proteins, most of them with still unknown function. Other GO terms found in this translation class are cell cycle process (GO:0022402), cellular component movement (GO:0006928) and response to stress (GO:0033554). The translational induction of some representative mRNA of this class such ATF4 and EGR2 was also confirmed by qPCR (Figure 4B).

### Translation vs. Transcription Changes Upon Stress

**Figure 5:**
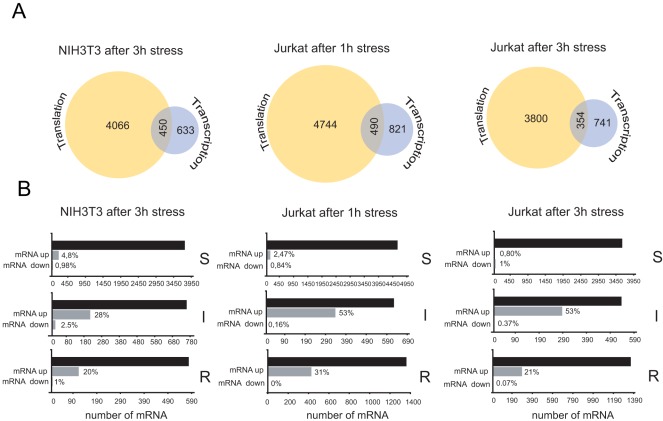
Analysis of stress-induced coordinate changes in translatome and transcriptome. A) Venn diagrams of mRNA that only experienced translational changes upon stress (log_2_ P/M change≥1 or≤−1, yellow circle), those that only experienced transcriptional changes (A change≥1 or≤−1, blue circle) or whose that significantly changed in both log_2_P/FM and A values (merged). The number of mRNAs for each group is shown. B) Changes in mRNA abundance for each translational class after stress. The number and percentage of mRNA whose abundance changed after stress (up or down) for each translational class (S, R and I) are shown in gray rectangles. Black rectangles show the number of mRNA that comprises each class based on their translational behavior upon stress. Note that a significant percentage of mRNA whose translation resisted or was induced by stress also increased in abundance, especially in Jurkat cells.

In yeast, transcriptional and translational changes for most mRNA upon stress are coordinated in the same direction to potentiate the general stress response in this organism [43], [68]. We therefore analyzed if a such as pattern was also evident in mammalian cells. Only 1,083 mRNA in NIH3T3 and 1093 mRNA in Jurkat cells changed significantly in abundance after 3 h of stress, in contrast to 4,500–5,300 mRNA that experienced a significant translational change under these circumstances (Figure 5A). Interestingly, 90% of these 1,083–1,093 mRNAs changed by increasing their abundance after stress. Functional analysis of these group of mRNA showed a specific enrichment in response to protein stimulus (GO:0051789, p<2×10^−4^) and unfolded protein (GO: 0006986, p<5×10^−2^) as expected [49], [69]. Venn diagrams showed that only a small fraction of total mRNA (450 in NIH3T3 and 354 in Jurkat) changed simultaneously in both translation and mRNA abundance after 3 h stress, in contrast to that found in yeast where about 70% of mRNA that changed translation after severe stress also changed in mRNA abundance [43], [68]. When we focused on specific translation classes, however, the coordination in translation and transcription changes was much more apparent. Thus, 20% of mRNA grouped into the translation arrest resistant class (R) and 28% of mRNA grouped into the translation inducible class (I) increased in abundance in NIH3T3 cells after stress (Figure 5B). This effect was even more apparent in Jurkat cells, where 53% of I class mRNA and 20–30% of R class mRNA increased in abundance after stress. This coordination, however, was not observed in translation sensitive (S) mRNA where only 3–5% of these mRNA increased in abundance after stress (Figure 5B).

We next focused on translation changes in mRNAs whose abundance increased after stress (>2 fold). In NIH3T3 cells (989 mRNA), 21.5% of these mRNA increased in translation upon stress and up to 18% experienced a significant translational repression. In Jurkat cells (1,020 mRNA), 22% of these mRNA increased in translation upon stress and only 3% were translational repressed.

### Features in mRNA that Define the Translation Classes

With the aim to find specific features in R and I classes of mRNA that could explain their translation during stress, we analyzed some parameters in sequence and structure that were reported before to influence translation initiation of specific mRNAs. Interestingly, translation inducible (I) class of mRNA in both mouse and human encode proteins nearly twice as large as the median of all protein coding mRNA detected in the chips. This feature was also apparent for R class of mRNA in Jurkat, but not in NIH3T3 cells. Another prominent feature found in I class mRNA was the prevalence of uATGs in the 5′ UTRs (Table 1). Thus, 36% and 24% of these mRNA in NIH3T3 cells showed at least 2 or 3 uATGs, respectively. Notably, the probability to find 2 or more uATGs in I class mRNA of NIH3T3 cells was 5–10 fold higher than in the group of mRNA with high translation rates and only slightly higher than in the group of mRNA with low translation, supporting the fact that I class mRNA are a particular type of low translation mRNA whose translation was relieved after stress. Another feature found in I class of mRNA was a lower G+C content in the 5′ UTR (60–61% vs 65–66% as median in all mRNA) and in the CDS region (49–50% vs 53% as median in all mRNA). An interesting observation was that, contrary to that found in NIH3T3 cells, mRNA with the highest translation scores in Jurkat cells contain uATGs in their 5′UTR, reinforcing the notion that these cells show a remarkable dysregulation in translation control. We also found that R class of mRNA was much more larger in Jurkat than in NIH3T3 cells, whereas for I class mRNA we found the opposite (Table 1). This result, together with the fact that R class mRNA showed a similar CDS length than I class mRNA in Jurkat cells, suggests some decanting effect from I class to R class in this cell line. Thus, we found many mRNA of R class in Jurkat whose corresponding ortholog in NIH3T3 cells fell into the I class (Figure S4).

## Discussion

The wide range of translation efficiencies reported before for yeast mRNA transcriptome has also been found here for mammalian mRNAs, ranging from low polysome-associated mRNAs to those that showed a high enrichment (>90%) in the polysome fraction such as some mRNA that encode components of proteasome or metabolic enzymes. Although abundant mRNA in NIH3T3 and Jurkat cells tended to show high translation efficiencies, this correspondence was only a trend and not a statistical significant correlation (R^2^<0.1). Thus, a prominent group of mRNA including those that encode some ribosomal proteins (e.g. RPS2 or RPS12) and some elongation factors (e.g. EEF1G) displayed low translation efficiencies despite were abundant messages, showing that for these mRNA subsets the regulation of protein outputs are mainly controlled at translation level as described before for specific mRNA [5], [6], [7], [70]. We found here that NIH3T3 and Jurkat cells, despite their differences, share a basic translatome encoded by the most abundant messages with a number of coherent biological themes. We also detected abundant lineage-specific mRNA with high translation efficiencies such as mRNA encoding the antigens CD3 or CD47 in Jurkat cells. For low expression mRNAs, the lack of correlation in translation efficiencies found between NIH3T3 and Jurkat probably reflects differences in the activity or regulation of one or more initiation factor(s) due to differences in lineage, species or degree of transformation. Thus, the tumorigenic nature of Jurkat cells probably explain the higher scores in translation efficiencies found for thousands of mRNA in this cell line. The absence of breaks that attenuate translation or the higher abundance (and/or activity) of some eIFs such as eIF4F, DHX29 or eIF2B [51], [52], [71], [72] found in many tumor cell lines could explain the elevated translation rates of many mRNA orthologs in Jurkat as compared with NIH3T3 cells.

Among the features in mRNA that could influence translation in eukaryotic cells, our analysis points out to the length of CDS, 5′ UTR, 3′ UTR and the presence of uORFs. The strong bias that high translation mRNA show for having shorter CDS has been reported before in yeast [19], [42] and corroborated here for mammalian cells. Abundant proteins involved in the basic functions of the cell probably compacted their mRNA to reduce ribosome transit times that accelerated translation, lowering at the same time the chance for any eventual ribosome misreading of mRNA during the decoding process. This idea has been recently supported by the low rates of protein evolution (purifying selection) found in highly expressed genes [73]. A short CDS length, together with a slightly better AUG context and a shorter 5′ UTR and 3′ UTR could explain the high translation rates for a subset of mRNA in NIH3T3 cells (Table 1). However, taken per separate these features do not necessary define a translational class; for instance, we found that low translation mRNAs are also characterized by a slightly shorter CDS than the median in both NIH3T3 and Jurkat cells.

Although far from perfect, the correlation found between mRNA levels and translation rates in yeast seems to be higher than in mammalian cells [43], [68], [74]. This probably reflects a much closer coordination between transcription and translation in yeast than in mammalian cells, especially when changes in mRNA abundance and translation were compared upon stress. Contrary to that found in yeast, we describe here that the changes in gene expression during the acute phase of stress response in mammalian cells were mainly translational, so that changes in translation dominated over those that affected transcription or mRNA stability. Unlike yeast that directly face environmental stresses in a unicellular manner and through a single eIF2 kinase (GCN2), stressful situations in mammalian cells are generally buffered by the homeostatic control of tissues and organs, so that a rapid translational response could be enough to cope with the acute phase of stress. However, a partial but clear potentiation effect in transcription and translation changes was observed for the subsets of mRNA that are translating upon stress in mammals (e.g. ATF4 and BiP), an observation that further strengthens the key role of these proteins in the stress response.

The presence of uATG in 5′ UTR is a distinctive feature of mRNA with low translation rates, especially for those whose translation was relieved after stress. Notably, we found here a relative abundant group of mRNA whose translation might be regulated by an ATF4-like mechanism. The finding that this group of mRNA was highly enriched in transcription factors (TF) of bZIP and zinc finger C2H2 classes supports the existence of a conserved programme that initiates with the rapid accumulation (via translational activation) of TFs that further induce a number of effector genes involved in the integrated response to stress [13], [24], [75]. In fact, a considerable number of these TFs are classified as early response genes (ERGs) whose expression changed rapidly after different stressful situations [49]. This coordination was also found among TF of different families that physically interact or whose expression can be integrated in networks [76]. Interestingly, most of TF mRNA whose translation was induced after stress has been classified as facilitators (AP1, ATF4, etc.) that positively regulated transcription in response to multiple stimuli in a wide range of tissues and organs [76]. Moreover, inducible class was also enriched in mRNAs that encode zinc finger C2H2 type-containing domains of known (e.g. EGR1, EGR2 or SP1) and yet unknown functions, some of the last are also probably involved in transcriptional regulation. Some effector genes induced by master TFs such as CHOP, ATF3 and GADD34 (regulated by ATF4) or COX2 (regulated by NFAT5) were activated at both transcriptional and translational levels after stress, showing the existence of a translational coordination network among mRNA encoding master regulators and some effectors of stress response. However, for other genes that were induced during the first hours of stress, translation of their mRNA probably required a later recovery of cellular translation promoted by eIF2 dephosphorylation, since these mRNA do not contain any autonomous mechanism of translational resistance to eIF2 phosphorylation [23]. This agrees well with the existence of two waves of gene expression response to recover from stress, the first (and immediate) dominated by translational changes, and the second wave where the products of many induced genes are accumulated.

Although the presence of uAUGs is highly enriched in translation inducible mRNAs, the long 5′ UTR of some these mRNA such as EGR2 and JUN do not show any uAUG (Figure S3). This, however, does not necessarily mean that translation regulation of these mRNA does not rely on the activity of uORFs, since recent data from RNAseq has revealed the existence of non-AUG uORFs that might be also operating in some yeast and mammalian mRNAs [42], [77]. Alternatively, stress-induced translational relief of some mRNAs that do not show any predictable uORFs might be relay on another sequence(s) or structural motif(s) in mRNA whose existence should be tested in the future.

The finding that translation arrest resistant mRNA are enriched in terms such as aminoacyl-tRNA synthetase (AARS), molecular chaperones, positive regulators of DNA replication and repair, and negative regulator of apoptosis reinforce the pro-survival nature of stress response. Adaptive changes in aminoacid metabolism during response to ER stress has been described before and was interpreted as an anticipatory response of the cell to recover translation after stress [69]. Our finding that many AARSs are synthesized during the acute phase of stress response supports the above notion. However, unlike the translation inducible transcripts, mRNA of the R class do not show any apparent anatomical feature except for a larger 5′ UTR of slight lesser content in G+C than the median of all mRNA. Some of R class mRNA such as BiP, HSP70 and HSP90 [78], [79], [80], and other I class mRNA such as MYC and JUN [81], [82] have been reported before to translate via an IRES element, although some recent analysis have seriously questioned the role of such elements in translation of cellular mRNAs [83], so that any direct association between translational resistance to stress and IRES activity will require a more careful analysis.

Finally, the current advances in deep sequencing (RNAseq) and high-throughput analysis of RNA structure by chemical/enzymatic probing and computation will help to identify elements of sequence/structure (RNA motifs) involved in translation regulation of mRNA subsets with coherent biological functions. In particular, the presence of specific structures downstream the AUG in mRNA that could allow eIF2-independent translation during stress as described before for some viral mRNA [62] will deserve further analysis.

## Materials and Methods

### Cell Culture

Low passage NIH3T3 (from ATCC, CRL-1658) cells were grown on 100 mm plates in DMEM supplemented with 10% of calf serum (Sigma). Jurkat cells (from ATCC, TIB-152) were grown in suspension in RPMI supplemented with 10% foetal calf serum (Gibco BRL). Cell cultures were treated with 10 µM of thapsigargin (Sigma) for the times indicated, washed with cold PBS and further processed for polysome extraction.

### Polysome Profiling and RNA Extraction

Four p100 plates of subconfluent NIH3T3 cells and 10^8^ Jurkat cells were used for the experiments. Five minutes before the lysis, cells were incubate with 50 µg/mL of cycloheximide (CHX) in order to freeze the polysomes. Cells were washed twice with cold PBS-CHX and lysed in polysome buffer (Hepes 30 mM pH 7.4, 100 mM KCL, 5 mM MgCl_2_, 1 mM DTT, 1% Triton X-100, 0.1% deoxycholate) supplemented with 0.5 mg/mL of heparin and 50 µg/mL of cycloheximide. After 15′ of incubation, cells were centrifuged at 8000 xg in a minifuge at 4°C. Supernatant was immediately loaded on 15 mL ultracentrifuge tubes (Beckman) containing a 10–40% sucrose gradient in polysome buffer. Polysome gradients used in this work were prepared all at once in a gradient marker machine (BIOCOMP) for reproducibility. Gradients were spun at 39,000 rpm for 3 h in a SW41 rotor (Beckman) at 4°C. Tubes were fractionated from the top using a ISCO fractionator apparatus to get 12 fractions that were extracted immediately with phenol-chloroform and precipitated with 2 volumes of ethanol overnight at −20°C. The RNA content of each fraction was analyzed by agarose electrophoresis. Fractions from 1 to 7 (free+monosome, FM) and from 9 to 12 (polysome, P) were pooled and precipitated with 1.5 M LiCl overnight at −20°C. This step was critical to eliminate any rest of heparin that inhibited the retrotranscription during the labeling process. The RNA pellet was washed with 70% EtOH and resuspended in lysis buffer of RNasy kit (Qiagen) and purified according to the manufacturer. Three independent polysome preparations for each cell type and condition were performed. Given the high reproducibility in the separation, the corresponding fractions from these three preparations were pooled and stored at −70°C to further used as material in the labeling reactions. Polysome fractions contained 2 fold (Jurkat) or 1.5 fold (NIH3T3) more total RNA than the free+monosome fraction in control cultures, whereas upon stress the free+monosome fraction accumulated 3 fold more RNA in both cell types. These proportions were kept for RNA labeling.

### Microarray Hybridization

RNA labeling and microarray hybridization was carried out according with the platform of two color microarray-based gene expression analysis 6.0 from Agilent. Briefly, RNA was labeled using the low Input Quick Amp labeling kit (p/n 5190–2306 Agilent) so that total RNA (FM+P) for every sample was kept to 300 ng. For instance, in unstressed Jurkat sample, 101 ng of FM RNA and 199 ng of P RNA were used for labeling reactions. This rendered 5 pmol/mL of Cy5-labeled FM cRNA and 6.55 pmol of Cy3-labeled P cRNA. Equal volumes (4 µL) of FM and P cRNAs were used for hybridization of dual color chips of 44 K from human (G2519F-014850) or mouse (G2519F-014868), and scanned on Agilent Scanner G2505B US45102947. Similar results were obtained when the same amount of FM and P RNAs was used for labeling, followed by the appropriate adjustment to the initial proportions during the hybridization step.

### Data Processing

All basic data processing was carried out using the tools suited in Babelomics 4 (www.babelomics.org, [57]). Raw data extracted from Agilent software 10.7.1 was loaded, background corrected (Normexp for Jurkat, Half for mouse) and the replicates of each mRNA sequence were averaged. Next, data were filtered by A values above 4 for Jurkat and 4.5 for mouse. A values are the mean of the log_2_-scaled intensities in green and red channels (½ log_2_ (RG)) and represent the relative abundance of each mRNA (FM+P). For some analysis, a more restrictive A cut was used. All the steps in data processing were checked twice to minimize the loss of information. The data discussed in this publication have been deposited in NCBI’s Gene Expression Omnibus and are accessible through GEO Series accession number GSE36206 and GSE36207. Basic and advanced statistical analysis were performed in Excel 2004 and in JMP8 software, respectively. A list with unique Ensembl transcript ID annotation was created for mouse (11,796 items) and human (21,917 items). The human-mouse ortholog list was extracted from MGI database (www.informatics.jax.org) and contains 17,861 ortholog pairs. Translation efficiency was defined as the ratio (in log_2_ scale) of mRNA abundance in polysomal (P) and non polysomal fractions (FM). To estimate translation changes for each mRNA, the log_2_P/FM value after stress was subtracted to log_2_P/FM value in control conditions. Functional analysis of translation classes was carried out by single or set enrichment analysis using FatiGO and FatiScan programs suited in Babelomics 4. Equilibrium base-pairing probabilities (BPPs) in mRNA were calculated via McCaskill’s partition function algorithm with the aid of Vienna RNA package (http://rna.tbi.univie.ac.at/). Mean BPP values in positions within UTR-CDS segment neighboring to either start or stop codons were calculated for different mRNA samples as described previously [58]. The information to construct the dataset of abundant proteins in 3T3 and Jurkat cells was extracted from previous reports [64], [65], [66], [67] and from http://www.meduniwien.ac.at/proteomics/database/#simple_search/.

### Quantitative PCR (qPCR)

RNA from P and FM fractions were retrotranscribed to cDNA using the Super Script RT II kit (Invitrogen) and random primers (Promega). Volumes of RNA used as templates were adjusted so that the total amount of FM+P was set to 1.5 µg for all the samples, keeping the ratios FM/P as describe above. For qPCR, a 1.5 µL sample of cDNA reactions was used. The primers were designed to target conserved regions in human and mouse, so that each primer pair can be used for amplification in both species (Table S2). Amplifications were carried out in a LightCycler® 480 apparatus (Roche) using the SYBR green I method (Roche). Most of amplifications were carried out using 0.5 µM of primers, a preincubation step at 95°C for 10 s, and 45 cycles of amplification consisting of a denaturing step at 95°C for 10 s, a annealing step at 55°C for 20 s and an extension step at 72°C for 30 s. To quantify EGR2 and Lig1 mRNAs, primer concentration was reduced to 0.1 µM and the annealing temperature was increased to 58°C. The apparatus was set for relative quantification.

### Metabolic Labeling and Immunoblot

Cells growing in 24 well plates were labeled with [^35^S]-Met for 30 min, washed twice with cold medium, lysed in buffer sample and analyzed by SDS-PAGE followed of autoradiography as described previously [62]. Immunoblot against phospho-eIF2α was carried out exactly as described previously [62].

## Supporting Information

Figure S1 **Polysome preparation of NIH3T3 cells and correlation between translation efficiencies and mRNA abundance.** A) Agarose gel analysis of RNA in fractions of polysome gradients from NIH3T3 cells under normal and stress conditions (10 µM thapsigargin treatment for 3 h). B) Correlation between translationefficiencies (log_2_P/FM) and mRNA abundance (A value) in NIH3T3 and Jurkat cells. Poor correlation coefficients (R2) were obtained.
(EPS)

Figure S2 A) Other cell-specific GO terms found in mRNA with high translation rates. B) Comparation of positional distribution of BBPs among maximal and minimal translation mRNAs in NIH3T3 cells. Initiation and termination codons are colored in green and red, respectively. +1 corresponds with the “A” of the AUG initiation triplet. At the stop triplets, +1 is the first letter of the codon (U). Significant differences (p<0.05) are in bold. Note the +18–26 stretch in the CDS and the +13–15 stretch in the 3′UTR that were significantly enriched in high translation mRNA.
(EPS)

Figure S3 A) Stress-induced translational activation of mRNA encoding transcription factors (TFs) in NIH3T3 and Jurkat cells. B) Network of physical and functional interaction among some TFs whose translation was induced by stress. Black lines show physical interactions, whereas green lines show participation in the same pathways. Only curated information from GeneMania (www.genemania.org) and String 8.3 (http://string-db.org) were used to construct the network.
(EPS)

Figure S4 **Translational dysregulation in Jurkat cells. Most I class mRNA in NIH3T3 fell into the R class in Jurkat cells.** We selected 651 mRNA of I class in NIH3T3 (control log_2_P/FM≤0+stress-control log_2_P/FM≥1) and found the corresponding ortholog in Jurkat cells for 554 mRNAs. The log_2_P/FM values for each class (n = 554) under both control and stress conditions were represented in boxplots with the mean as a red line.
(EPS)

Table S1 **Translation efficiencies of mRNA encoding some abundant proteins in NIH3T3 and Jurkat cells.** The abundance of these mRNAs (A value) is also shown.
(XLS)

Table S2 **List of primers designed to amplify both human and murine mRNAs by qPCR.** The hybridization sites in human and murine RefSeq mRNAs are shown.
(XLS)
